# The intake pattern and feed preference of layer hens selected for high or low feed conversion ratio

**DOI:** 10.1371/journal.pone.0222304

**Published:** 2019-09-12

**Authors:** Cameron E. F. Clark, Yeasmin Akter, Alena Hungerford, Peter Thomson, Mohammed R. Islam, Peter J. Groves, Cormac J. O’Shea

**Affiliations:** 1 School of Life and Environmental Sciences, The University of Sydney, Camden, NSW, Australia; 2 Poultry Research Foundation, Sydney School of Veterinary Science, The University of Sydney, Camden, NSW, Australia; 3 School of Biosciences, University of Nottingham, Loughborough, England, United Kingdom; Universidade Federal de Viçosa, BRAZIL

## Abstract

Feed accounts for the greatest proportion of egg production costs and there is substantial variation in feed to egg conversion ratio (FCR) efficiency between individual hens. Despite this understanding, there is a paucity of information regarding layer hen feeding behaviour, diet selection and its impact on feed efficiency. It was hypothesised that variation in feed to egg conversion efficiency between hens may be influenced by feeding behaviour. For this experiment, two 35-bird groups of ISA Brown layers were selected from 450 individually caged hens at 25–30 weeks of age for either low FCR < 1.8 ± 0.02 (high feed efficiency (HFE) or high FCR > 2.1 ± 0.02 (low feed efficiency (LFE)). For each of these 70 hens, intake of an *ad-libitum* mash diet at 2-minute time intervals, 24 h a day, for 7 days was determined alongside behavioural assessment and estimation of the selection of components of the mash. The group selected for HFE had a lower feed intake, similar egg mass and associated lower FCR when compared with the LFE group. Whilst feed intake patterns were similar between HFE and LFE hens, there was a distinct intake pattern for all layer hens with intake rate increasing from 0300 to 1700 h with a sharp decline to 2200 h. High feed efficiency hens selected a diet with 25% more ash and 4% less gross energy than LFE hens. The LFE hens also spent more time eating with more walking events, but less time spent resting, drinking, preening and cage pecking events as compared with HFE hens. In summary, there was no contrasting diurnal pattern of feed consumption behaviour between the groups ranked on feed efficiency, however high feed efficiency hens consumed less feed and selected a diet with greater ash content and lower gross energy as compared with LFE hens. Our work is now focused on individual hen diet selection from mash diets with an aim of formulating precision, targeted diets for greater feed efficiency.

## Introduction

Feed efficiency (FE) is an important production trait as feed accounts for 60–70% of the costs for layer production systems [1, 2]. Reducing bird feed requirements through reduced feed intake requirements while maintaining egg mass would substantially improve the profitability of poultry production systems. In this regard, appetite, egg mass, and body weight dynamics are the most important traits involved in the variation of laying hen feed intake [3, 4] and these traits can be used to predict feed consumption from multiple linear regression [5]. Laying hens can store an excess of energy through egg yolk and body fat [1]. Some differences in heat production and thus FE have been attributed to differences in behaviour between birds. In this regard, hens showing more locomotor activity have greater heat production [6] and/or lower efficiency of feed utilisation [7]. In laying hens, an increase in heat production was associated with specific behaviours such as standing, running, feeding, drinking and preening [8]. How such feeding behaviours change over time and their levels in high or low FE commercial birds are unknown.

Feed intake pattern in laying hens is influenced by the egg production cycle [9]. Where a mash is offered rather than a complete pellet or where whole grains or calcium are offered as ancillary supplements, hens may select different components of the diet at specific times of the day in line with nutrient and energy demands during egg formation [10]. Increased feed intake immediately after oviposition may reflect an increased need for amino acids required for secretion of albumen [11], while Molnár et al. [12] reported no evidence for an hour to hour regulation of feed intake, apart from for calcium. As calcium content of a diet is a key driver for the regulation of hen intake when complete feeds are offered targeting a set energy intake, hens may overconsume energy to satisfy calcium requirements, or conversely, when mash diets are offered specifically select out calcium from the mixed diet substituting the energy fraction. In a recent study [4], it was demonstrated that ISA Brown layers housed under common environmental conditions and offered a common, ad libitum mash diet show considerable variation in voluntary feed intake, comparable egg mass, and consequently considerable variation in feed to egg conversion ratio (FCR). However, there is a paucity of data on the link between nutrient selection from complete mash diets and feeding behaviour and FE for contemporary commercial laying hens. Therefore, the objectives of this experiment were to determine the behaviour, intake pattern and feed preference of high or low FE ISA Brown layer hens ranked based on long term FE status when offered a complete diet in a mash form. Thus, we hypothesised that birds of high FCR when mash diets are offered hens may specifically select a diet with greater calcium fraction as compared with low FCR birds and would also have reduced locomotor activity.

## Material and methods

### Experimental design and animal management

This work was conducted at the University of Sydney, Poultry Research Facility, Camden, Australia using an initial pool of 450 ISA Brown hybrids birds (25 weeks of age). All experimental procedures conducted in this study were approved by the University of Sydney Animal Ethics Committee (Project Number 2017/1212) and were in accordance with the Australian code for the care and use of animals for scientific purposes (8th Edition, National Health and Medical Research Council, 2013). Birds (49 weeks of age) were randomly selected and housed individually in 25 × 50 × 50 cm cages for an initial screening period of 6 weeks with a 14 h lighting program from 0600 to 2000 and 10 h of darkness to facilitate subsequent ranking of birds based on FCR (FCR; weekly feed intake ÷ weekly egg mass output). Individual weekly feed intake, daily egg production and egg weight were recorded. The dietary composition, ingredient and nutrient composition are provided in Table 1.

**Table 1:** Dietary composition of the experimental basal diet.

Feed ingredient	Amount (kg/tonne)
Wheat 10.5%	347
Sorghum 12%	345
Soybean meal 46%	155
Limestone Grit 38%	71.0
Canola Expel 32%	30.0
Limestone	20.0
Dicalcium phosphate	15.0
Soy oil	7.00
Sodium bicarbonate	3.19
DL-methionine	1.75
Lysine-HCL	1.70
Salt	1.60
Layer Premix	1.00
L-Threonine	0.45
Choline Chloride 60%	0.30
Ronozyme WX CT (DSM)	0.12
Ronozyme Hi-phosphate Layer 300 (DSM)	0.03
Total	1000
**Calculated nutrient analysis**	
Metabolisable energy (kcal/kg)	2750
Crude protein%	16.3
Total digestible lysine%	0.74
Total digestible methionine%	0.39
Total digestible tryptophan%	0.18
Total digestible isoleucine%	0.58
Total digestible arginine%	0.83
Total digestible valine%	0.65
Crude fat%	2.90
Linoleic acid %	1.39
Calcium%	4.00
Total Phosphorus (P)%	0.61
Available P%	0.40
Sodium%	0.18
Crude ash%	13.4
Lysine%	0.82
Methionine%	0.42
Xantophyll (mg/kg)	6.00

**Fig 1:**
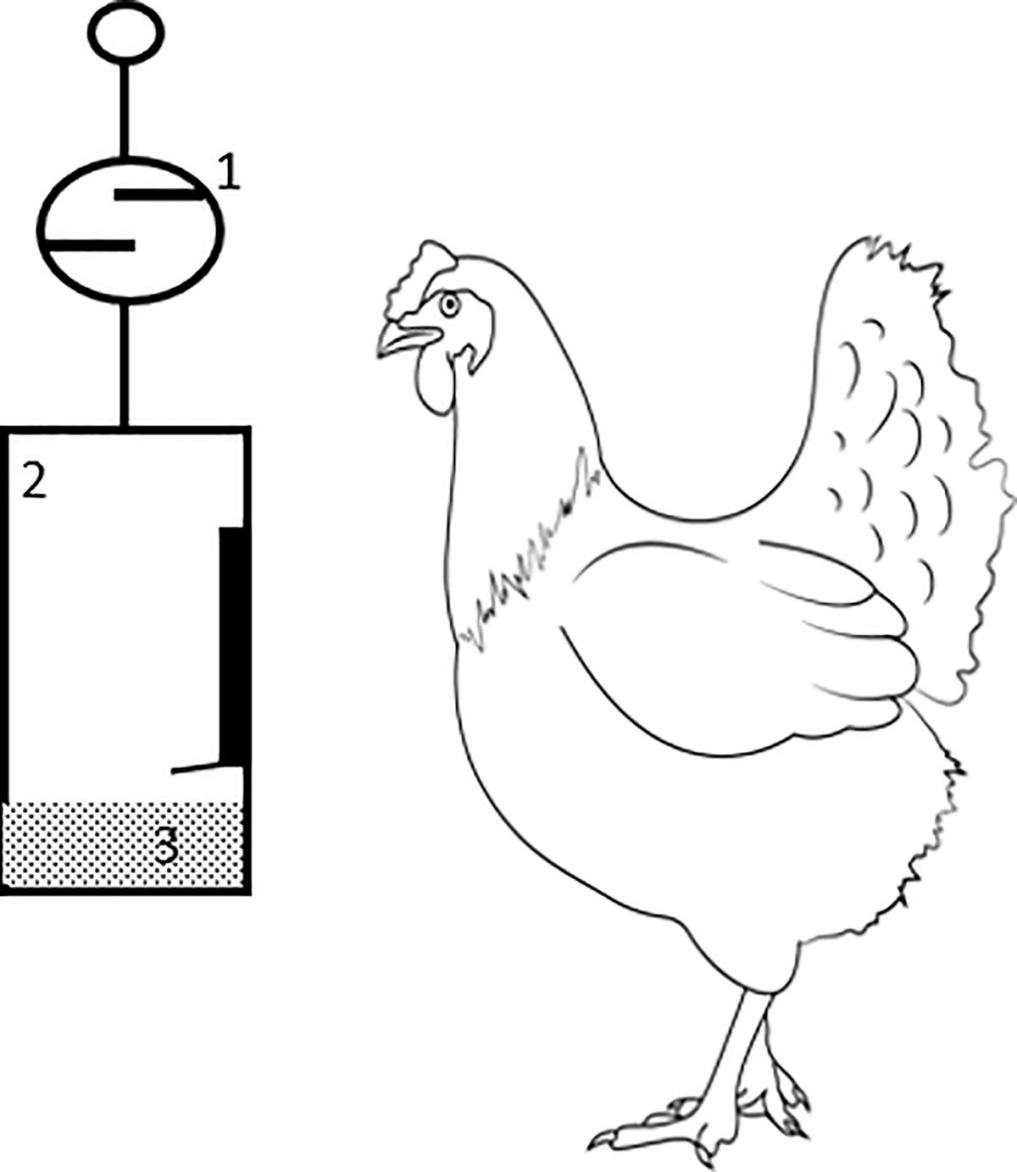
Scale system with layer hen; 1 –wireless analogue ‘pull’ load cell sensor; 2—bottle with opening and lip to prevent feed spillage; 3—feed.

Eggs were collected daily, weighed using a digital scale and the average egg weight was determined per hen. Egg mass per hen per day was calculated as laying percentage, multiplied by average daily egg weight. Feed conversion ratio was calculated from weekly egg mass production and weekly feed intake over 6 weeks to verify the feed efficiency of each group. At the end of experiemental period, all 450 birds were ranked and grouped based on their overall mean FCR. Birds with an FCR of ≤ 1.80 ± 0.01 were grouped as high feed efficiency (HFE), whilst birds with FCR < 2.02 ± 0.01 and FCR ≥ 2.31 ± 0.01 were grouped as medium feed efficiency (MFE) and low feed efficiency (LFE). In total, 150 birds (35 weeks old) were selected for the second phase of the study, Groups were monitored for individual FI, EP and EW for a further 6 weeks to allow confirmation of the continuing FE status of individual birds. At the end of the experimental period, all 150 birds were ranked and grouped based on their overall mean FCR. Feed conversion ratio of HFE hens was ≤ 1.78 ± 0.02 whilst in MFE and LFE hens this values were ≤ 1.91 ± 0.02 and ≥ 2.08 ± 0.02 respectively. The top 35 high feed efficiency (HFE; FCR < 1.8) and bottom 35 low feed efficiency (LFE; FCR ≥ 2.1) hens (49 weeks of age) were selected for a feeding behaviour study of 10-week duration. Hens were randomly housed singly and within visual and auditory proximity to each other regardless of FE ranking. A total of 14 hens (7 HFE and 7 LFE) were then monitored in the first period for intake every 2 minutes of 24 h for 7 d following 7 d of adaptation using a prototype hanging scale system (G7 wireless analogue sensor range 0-5kg; Ease Mind Technology Ltd., Hong Kong) (Fig 1). All eggs were collected from each group and weighed daily. The oviposition time of each individual hen was recorded, from 08:00 to 10:00 h at 0.5 h intervals. After this period, a new group of 14 hens were monitored in the same way until all birds had been monitored over 5 periods. Feed samples were collected daily at the time when feed was offered to determine the nutrient composition of feed on offer. Feed remaining for each bird at the end of each 7-d monitoring period was analysed for ash, nitrogen and gross energy to determine the propensity of birds to select individual components of the mash diet. For the behaviour study, each hen was video recorded for 1 h in order to characterise general activity and feeding behaviour. Video recording was during the light portion of the photoperiod at the normal illumination intensity in the room. Four cameras were operated simultaneously in order to record all birds on the same day between 1100 h and 1200 h with each camera viewing 4 adjacent cages housing birds. Routine feeding, and egg collection were carried out on days during which video recording of behaviour took place. The behavioural states [13] recorded were as follows: eat, still, rest, head flick, preen, drink, walk, cage pecking, feeder pecking, preening.

### Dietary analysis

The gross energy of the common experimental diet and refused feed were determined by an adiabatic bomb calorimeter using an adiabatic calorimeter (Parr 1281 bomb calorimeter; Parr Instruments Co.). Dietary nitrogen levels were determined using LECO Procedure (Leco Corporation, St Joseph, MI). The crude ash content of diet and refused feed were measured according to AOAC [14] procedures.

### Statistical analysis

Differences between consecutive weight observations every 2 minutes were calculated as an estimate of the amount of feed consumed over that interval by the bird (n = 324,119 weight differences). However, the daily addition of feed resulted in extreme weight differences, hence any weight difference more than five standard deviations from the mean were excluded from analysis. This process was repeated four times resulting in a data set of 320,837 differences. Difference data was then binned into consecutive 1h intervals, and the mean and standard deviation over each interval for each bird calculated. Mean values were multiplied by 30 to obtain total amounts of feed consumed over each 1h period. The mean data and standard deviations were used in subsequent analysis (n = 10,933 means and n = 11,024 SDs) after further extreme-value filtering, i.e. removal of values exceeding four standard deviations away from the mean of each data set.

For the total and standard deviations, the following linear mixed model was fitted to the data:

Y=constant+Group+Day+Hour+Group.Day+Group.Hour+Bird+ε,

where Y is the trait being analysed (total or SD); Group, Day, and Hour are fixed effects, with fitted interactions Group.Day and Group.Hour, and Bird is a random effect. The random errors ε were modelled using an ARMA (P = 1, q = 1) structure to allow for serial correlation between consecutive observations. The ‘lme’ function from the ‘nlme’ package in R was used for model fitting, and all analyses were undertaken using R.

As there were differences in feed remaining at the end of each experimental period due to differing intakes, any bird with feed remaining greater than one standard deviation from the mean was omitted from this analysis. Nine birds were omitted from HFE and nine birds were omitted from LFE. A generalised reduced gradient algorithm was used with the target of 0 for the difference in feed nutritive value remaining at the end of the experimental period and a diet where each of the feed types proportions could vary. This allowed the preference of each of the mash component feeds for each FE group to be estimated.

### Comparison of feeding system

Before the experiment, a preliminary study was conducted to ensure the daily intake for birds feeding from the metal feeder system used to screen the 450 birds for FE was the same as the scale system shown in Fig 1. Daily intake was monitored across 5 days for 7 high and 7 low feed conversion efficiency birds for both feeding systems. Linear mixed models were used for analysis with feeding system as a factor in the model and bird and day included as random effects. Results of this analysis showed the intake for birds between feeding systems to be similar (Mean 114g/bird/day; P = 0.55).

## Results

The crude protein, energy and ash of the main ingredients of the common wheat-soybean meal based mash diet offered to birds is presented in Table 2.

**Table 2:** Proximate analysis of the main ingredients used in the common wheat-soybean meal based mash diet offered to all hens.

Ingredient	Crude Protein%	Gross Energy (MJ/kg)	Ash%
Wheat	10	16	2
Sorghum	12	17	2
Soy	47	18	8
Lime grit	-	-	100
Canola expeller	35	20	8
Limestone	-	-	100
Dicalcium phosphate	-	-	85

**Fig 2:**
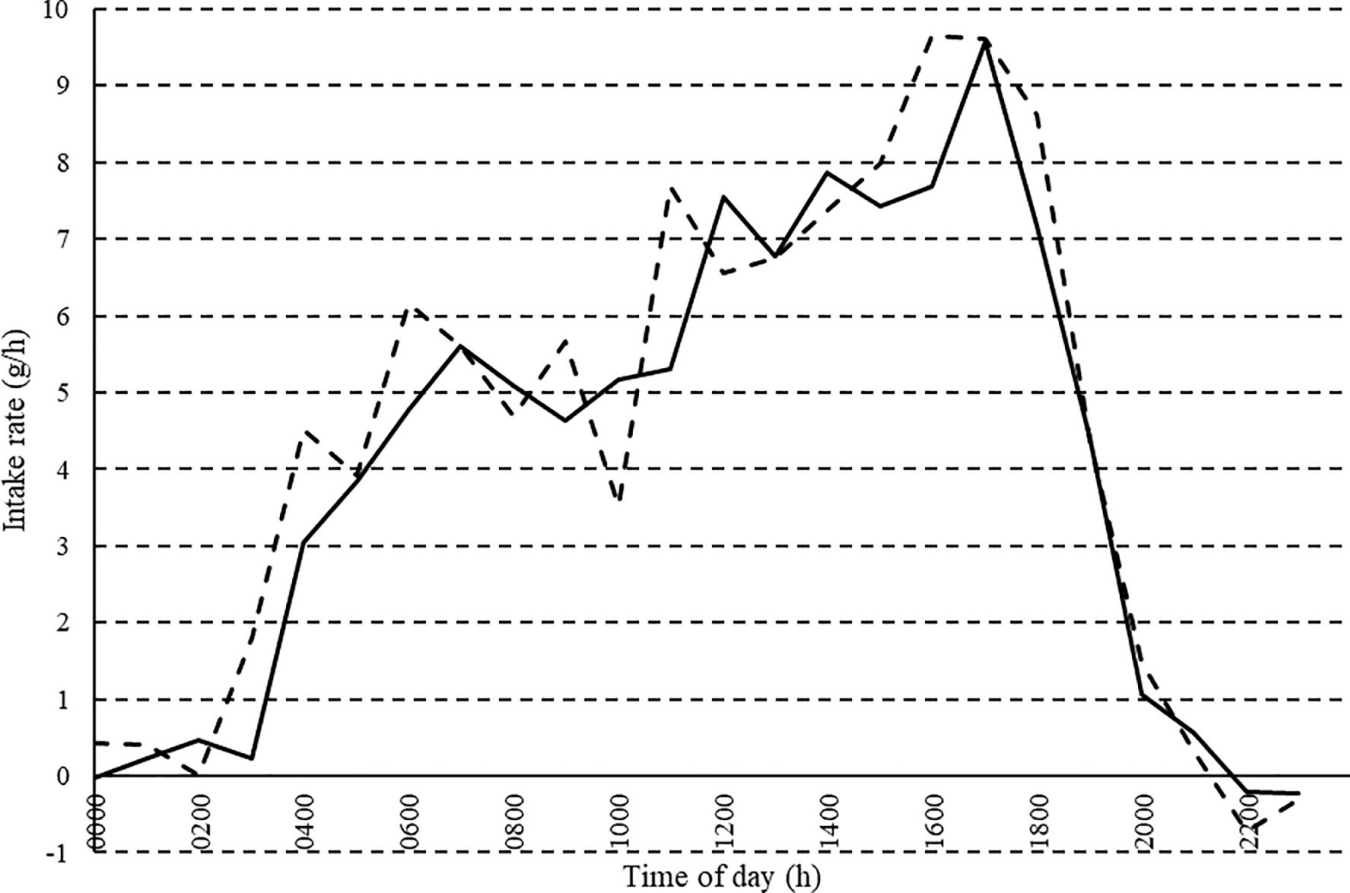
Predicted mean hourly intake rate (g/h) across 24 h for 35 HFE (solid line) and 35 LFE (dashed line) Isa Brown birds.

Feed intake (g/d), egg mass (g/d) and FCR during the experimental period are presented in Table 3. Average daily FI was greater in LFE birds (P *<* 0.001) and birds designated as HFE pre-experiment (25–30 weeks of age) continued to have lower FCR (P < 0.001) during the experimental period. Egg mass was similar (P > 0.42) between FE groups. There was no effect of FE group or day of study on intake patterns. However, there was an impact (P < 0.001) of time of day on intake rate (Fig 2) with both HFE and LFE birds steadily increasing feeding rate (g/h) from 0300 h to 1700h, after which intake rate linearly decreased to zero by 2200h.

**Table 3:** Feed intake, egg mass, feed conversion ratio and ash, nitrogen and gross energy remaining in the feed for HFE and LFE birds (49 weeks of age; n = 35/group).

Parameter	HFE	LFE	SED	*P-Valu*e
Feed intake (g/d)	120	136	2.5	<0.001
Egg mass (g/d)	65.6	63.5	1.5	0.42
Feed conversion ratio (FCR)	1.84	2.2	0.03	<0.001
Ash in remaining feed (%)	15.9	20.0	1.4	<0.01
Nitrogen in remaining feed (%)	2.6	2.5	0.45	0.18
Gross energy in remaining feed (MJ/kg)	14.2	13.6	0.25	0.07

HFE: High feed efficiency; LFE: Low feed efficiency

The nutritive value of feed remaining in the diet at the end of the experimental period for HFE and LFE birds is provided in Table 4. Crude protein remaining in the diet was similar between HFE and LFE birds, however, HFE birds selected a diet that had 25% more crude ash and 4% less gross energy than LFE birds. The HFE diet that remained in the troughs thus comprised less grain and more soybean meal, di-calcium phosphate and limestone.

**Table 4:** The estimated wheat and sorghum, soybean and lime and dicalcium phosphate composition (%) of the remaining diet for HFE and LFE birds.

Ingredient	HFE	LFE
Wheat and sorghum %	65	59
Soybean %	18	21
Lime and Di-calcium phosphate %	14	17

HFE: High feed efficiency; LFE: Low feed efficiency

The behaviour of the hens in this study is presented in Table 5. Feed efficiency had no effect on the number of feeder visits per h, hen head flicks/h, still or stand/h and feeder pecking/h. However, LFE hens had a greater number of walking events but less resting, cagepecking, drinking and preening events in comparison with HFE birds. Despite the similar number of feeders visit events between FE groups, the HFE birds spent less time feeding (21 minutes per h; P < 0.001) than the LFE birds (32 minutes per h).

**Table 5:** Number of behavior events recorded (number over a 1-hour period) for high feed efficiency (HFE) and low feed efficiency (LFE) layer hen groups at 49 weeks of age (n = 35/group).

Behaviour events (number/h)	HFE	LFE	SED	P—Value
Feeder visits	24	25	4.1	0.837
Time spent for feeding	21	32	3.0	<0.001
Head flicks	24	26	4.7	0.568
Rest	5	3	1.1	0.013
Still	11	9	2.8	0.336
Walking	12	20	1.9	<0.001
Cage pecking	20	17	1.5	0.024
Drinking	8	2	0.7	<0.001
Feeder pecking	15	12	2.8	0.268
Preening	19	14	0.8	<0.001

## Discussion

Variation in FE in ad libitum-fed, healthy contemporary hybrid caged layers were predominately influenced by variability in voluntary feed intake with egg mass being comparable between FE groups. Variation in maintenance energy expenditure was shown to be a major contributor to variation in residual FI between hens with similar egg mass production [15]. In this study, no contrasting pattern of feed consumption was observed for hens ranked as HFE when compared with LFE group, but differences in behaviour and ingredient selection from a mash diet were observed. Based on proximate analysis of remaining feed, it was observed that HFE group selected a diet with 25% more crude ash and 4% less gross energy than LFE group, with the HFE group comprising an estimated diet selection comprising less grain and more soybean, di-calcium phosphate (DCP) and lime. These results clearly show that given some ability to discriminate, the capacity for birds to select diets based on an appetite for minerals, protein and/or energy sources during the day to satisfy their own feed type preference and/or needs. Dietary ash content (mainly Ca) regulates intake [16] as hens tend to select a greater Ca intake when offered Ca separately, even when the feed offered is high in Ca and when Ca is offered separately from the other nutrients, the intake of each can be regulated independently of the other [17]. When hens are offered separate sources of energy, protein and Ca, hens can select Ca to meet their requirement with minimum protein and ME intakes to support high egg production [18]. Also, laying hens offered separate concentrated feed sources of either protein, energy or Ca, showed a more synchronised laying pattern with more eggs being produced early in the day compared with birds fed conventionally [19]. Lower ash consumption in LFE group could be due to a greater intake of energy whereas higher soybean consumption in HFE group may be related to an increased amino acid requirement for the synthesis of egg protein. Using the same experimental model, we previously reported that HFE group produced eggs with significantly greater albumen weight [4].

Despite the observation the LFE hens consumed more feed over the course of the entire day, the intake pattern was similar between FE groups in our work. In this regard, similar animals can show quite different levels of intake and dietary preference [20]. In the present study, a common, distinct intake pattern at an hourly level for all hens was observed, with intake rate increasing from 0300 to 1700 h and a rapid decrease in intake rate to 2100 h. Duncan and Hughes [21] showed feeding activity to decrease at the time of luteinizing hormone release at ovulation (6–8 h later), when the egg enters the shell gland, before oviposition with an increase in feeding activity following oviposition. In our experiment, birds started eating approximately 3 h before the lights came on at 0600 h and reached an initial intake rate peak between 0600 and 0700 h. This initial peak occurred 1–2 h before peak oviposition at 0800–0900 h. In line with our findings, Savory [22] and Kadono et al. [23] showed eating activity to decrease for 1–2 h before oviposition with intake rate increasing after this. The high intake rate after oviposition may firstly compensate for low intakes during oviposition, an increased demand for nutrients that occurs due to ovulation 30 minutes after oviposition and the birds demand for calcium which is greatest from early afternoon until late evening. This large increase in intake before lights were turned off may also allow the crop to act as a reservoir. Intake decreased to 2200 h when lights were turned off at 2000 h possibly suggesting the hens had some sense of day length, anticipated day end and modified their feeding behaviour by feeding at higher rates when the photoperiod begins. According to Khalil et al. [24], an implication of this anticipatory behaviour is ensuring enough feed supply to meet this increased FI before lights go off.

High FE hens spent less time feeding per hour (36%) as compared with LFE hens (54%). Hens which had a low actual feed intake relative to the average feed intake of the group (low residual FI hens) have been reported to have shorter feeding times per hour than high residual FI hens [25]. In the current study, LFE hens had similar levels of egg mass but increased walking and reduced resting (twice as much time) compared to HFE hens. Physical activity such as feeding, walking or locomotion at varying speeds is one of the most influential behavioural traits for intake in chickens [26, 7] and all these activities are associated with heat production [6]. Further, Luiting et al. [27] revealed that 80% of the genetic difference in residual FI between lines of chickens divergent for residual FI could be related to a difference in physical activity. Preening and cage pecking are considered as a comfort behaviour in birds [28]. Moreover, Broom [29], Cronin et al. [30], Henson et al. [31] demonstrated that cage pecking and feather preening are stereotypic behavioural adaptions to restraint, frustration or stress. Reduced cage pecking and preening with less resting in LFE hens suggests that inefficient hens may have been more occupied eating and walking with less associated time to develop stereotypic behaviours. In addition, HFE hens spent more time drinking relative to LFE hens in line with the findings of Marks and Pesti [32] who showed high FE birds to have a greater water intake and lower abdominal fat. Given our current findings related to diet selection, increased drinking in HFE birds is likely linked with the greater mineral content of the diet selected and associated greater effective osmotic pressure in plasma [33].

## Conclusion

Our work shows the ability of layers hens to regulate the intake of nutrients which can be used to increase feed efficiency. Greater levels of comfort behaviours such as resting, and preening shown by HFE birds contrast with greater levels of restless behaviour such as walking/pacing walking in LFE birds. Further studies are required to determine the mechanisms underlying layer birds feeding behaviour and its impact on production performance and FE. The impact of offering feed nutrient profiles to the entire flock according to HFE bird preference requires evaluation both for reducing feed costs and increasing egg quality.
